# Noise-Robust image edge detection based on multi-scale automatic anisotropic morphological Gaussian Kernels

**DOI:** 10.1371/journal.pone.0319852

**Published:** 2025-05-05

**Authors:** Lei Liang, Junming Chen, Jiawei Shi, Kai Zhang, Xiaodong Zheng

**Affiliations:** 1 College of Arts, Nanjing University of Information Science and Technology, Nanjing, China; 2 Faculty of Humanities and Arts, Macau University of Science and Technology, Macau, China; 3 Department of Visual Communication Design, Yeungnam University, North Gyeongsang, Korea; IIIT Kurnool: Indian Institute of Information Technology Design and Manufacturing Kurnool, INDIA

## Abstract

This paper presents a novel multi-scale, noise-robust edge detection method that employs multi-scale automatic anisotropic morphological Gaussian kernels to extract edge maps from input images. It addresses the issue of cross-edge detection failure in the Canny edge detector. Compared to other edge detection methods, the proposed approach offers significant advantages in maintaining noise robustness while achieving high edge resolution and accuracy. The paper is structured into five key sections. First, we propose a multi-scale automatic anisotropic morphological directional derivative (AMDD) to capture local gray-level variations around each pixel at multiple scales. Second, a new fused edge strength map (ESM) is introduced based on the multi-scale AMDD. Third, we analyze why the Canny isotropic Gaussian kernel detector fails to detect cross edges. Additionally, the edge contour is extracted by incorporating the fused ESMs and the edge direction map (EDM), which are processed through spatial and directional matching filters, into the standard Canny detection framework. Finally, we evaluate the proposed method using precision-recall (PR) curves and Pratt’s Figure of Merit (FOM). We compare its performance with existing state-of-the-art detectors on a standard dataset. Experimental results demonstrate that the proposed method effectively reduces noise, mitigates irrelevant signal interference, and smooths the image, showing competitive performance in edge detection tasks.

## 1 Introduction

The development of edge detection has spanned over half a century. In 1959, Julez [1,2] first introduced the concept of edge detection, opening the door to this vital field of study. In 1963, L.G. Roberts further advanced edge detection research by proposing the canonical Roberts operator [3]. Since then, edge detection has garnered significant attention and quickly became a central focus in image processing [4]. Although the Roberts operator, known for its fast computational performance, detects edges by subtracting diagonal or adjacent pixel values, it is susceptible to noise. Consequently, several improved versions of the Roberts operator were introduced, including the Sobel operator [5], Prewitt operator [6], and Kirsh operator [7]. While these operators offer some advantages, none are perfect, and each has its limitations. Subsequently, the concept of non-maximum suppression was introduced to address the issue of edge broadening in regions near image edges. This technique helps to refine edge localization by suppressing non-maximum responses, thus improving edge detection accuracy.

In the mid-1960s, based on the nonlinear characteristics of morphology, Lee Haralick and other researchers [8] proposed a fuzzy minimum morphological edge detection operator in 1987. Subsequently, Feehs-Arce introduced the *α*-adjusted edge operator [9], and Song Xudong et al. proposed selective ordered filtering methods [10]. These approaches successfully applied morphological techniques for noise suppression, utilizing various combinations of morphology and image features [11]. They effectively filtered irrelevant signals while preserving the inherent edge information. Building on the Canny isotropic edge detection method and existing multi-scale concepts, Zhang and Sun addressed the trade-offs between edge resolution, edge stretching, and noise stability [12–14]. They employed anisotropic directional derivatives (ANDDs), which combine small-scale isotropic Gaussian kernels (IGKs) with large-scale anisotropic Gaussian kernels (AGKs). The AGKs, due to their inclusion of rotation factors, can be considered as differential operators in conjunction with rotation windows. In addition, statistical methods were applied to detect geometrically deformed image edges using edge strength maps (ESMs) and edge direction maps (EDMs) derived from rotation windows [15]. Similarly, this rotating window concept has been successfully applied in synthetic aperture radar (SAR), where researchers demonstrated that it outperforms traditional differentiation-based edge detection methods [16,17].

Noise is not only introduced during the image transmission process but also arises in varying degrees due to operations such as first derivatives. Consequently, ANDDs are particularly sensitive to noise, especially impulse noise. To mitigate this, morphological methods such as the median filter (MF) and weighted median filter (WMF) [18] can be integrated with the ESM and EDM for noise reduction. Shui and Wang [19] proposed an anisotropic morphological directional derivative operator (AMDD), which leverages the relationship between the rotating double window attributes and the weighted median filter to effectively reduce impulse noise, thereby enhancing edge detection performance. In recent years, deep learning, a research area closely aligned with artificial intelligence, has experienced explosive growth, giving rise to numerous algorithms and models [20]. One of the most representative algorithms in edge detection is the Holistically Nested Edge Detection (HED) model [21].

The algorithm presented in this paper is based on ANDD and AMDD. This paper proposes a multi-scale omni-bearing automatic anisotropic morphological directional derivative (MAMDD) binding multiscale AGKs and morphological idea. MAMDD filter can extract local gray change information of images in multiple directions. The ESM and EDM extracted by small-scale ANDD have high edge resolution, accuracy, and little edge stretch but are sensitive to noise. Instead, large-scale extraction of ESM and EDM can counteract noise well, but the edge elongation effect is noticeable. Meanwhile, the edge resolution and accuracy are rather low. The reason why the three scales can be complementary in terms of performance attributes is that the mid-level scale extraction can well reconcile the edge resolution, accuracy, and robustness of noise. Therefore, a scale-fitting technique integrates edge maps from multiple scales to form a new ESM with excellent performance. At the same time, the EDM is extracted by AMDD matching filter, which embeds ESM and EDM into a standard Canny detector, and then a multiscale omnidirectional anisotropic morphological directional derivative (MAMDD) filter detector is constructed. The detector is compared with the existing state-of-the-art edge detectors regarding FODS, FOIS index, accuracy-recall, and quality factor FOM on the BSDS500 and Pascal VOC Challenge data set.

The paper is organized into four chapters, excluding the introduction. In Chapter Two, the concept of multi-scale and multi-directional anisotropic morphological directional derivatives (MAMDDs) is introduced, along with their discrete versions. The parameters and properties of these derivatives are analyzed, including edge resolution, edge stretching effects, and noise robustness. Chapter three presents a new edge detection method (ESM) and an edge detection model (EDM) and proposes a multi-scale, multi-directional anisotropic morphological directional derivative (MAMDD) edge detector. In Chapter Four, the performance of the MAMDD edge detector is evaluated with varying parameters, and the results are compared with those of state-of-the-art edge detectors across multiple performance indicators. Finally, the paper concludes with a summary of the findings.

## 2 Anisotropic morphological Gaussian Kernel and its directional derivative

In this chapter, the anisotropic morphological Gaussian kernels (AMGKs) and their corresponding derivative expressions are derived from the anisotropic Gaussian kernels (AGKs). The properties and characteristics of these kernels are then analyzed in detail.

### 2.1 Anisotropic Gaussian Kernels representation

The Canny edge detection operator is widely regarded as one of the most versatile among classic differential-based edge detection methods. It excels in terms of noise suppression and edge localization accuracy, delivering superior detection performance.

Within a given range of noise levels, the Canny algorithm employs a Gaussian smoothing filter, which is considered an optimal filter. A simple one-dimensional Gaussian kernel function is defined by the following formula (1).


gσ(x)=12πσ2 exp ⁡  (−x22σ2) (1)

According to the formula, *σ* is the standard deviation (also called scale) of the Gaussian distribution, and *x* is the distance from the origin on the horizontal axis. Firstly, the average value of the number of *n* was calculated. Then subtract the average from the *n* numbers to get another *n* numbers, which are different from the previous *n* numbers, square the other *n* numbers and add them up to obtain a number; finally, divide this number by *n* and extraction of the square root is *σ*.

The expression (2) of the Gaussian kernel function of size  ( *k* + 1 ) ( *k* + 1 )  can be obtained by extending the one-dimensional Gaussian kernel function to a two-dimensional plane.


gσ(x,y)=12πσ2 exp ⁡  {− ((x−(k+1))2+(y−(k+1))22σ2)} (2)

After selecting the optimal Gaussian smoothing filter, it is essential to determine the size of the Gaussian filter kernel, as the smoothing effect varies with kernel size. In general, most edge detection algorithms in the literature employ a 3x3 convolution kernel. The anisotropic Gaussian kernel function (AGKs) is obtained by compressing the two-dimensional Gaussian kernel on the x-axis with a certain ratio *ρ* and stretching it with the same ratio *ρ* on the y-axis is given by the following formula (3).


$$\begin{array}{ccc} g_{\sigma ,\rho }(u)=\frac{1}{2\pi \sigma^{2}}exp\left(-\frac{u^{T}pu}{2\sigma^{2}}\right),u=\left(\begin{array}{c} x\\ y \end{array}\right),u\in Z^{2}. & & \end{array}$$ (3)

In the formula (3), $p=\left(\begin{array}{cc} \rho^{2} & 0\\ 0 & \rho^{-2} \end{array}\right)$, and *ρ*  ( *ρ* ≥ 1 )  is the anisotropic factor. When *ρ* = 1, the expression (3) is an isotropic Gaussian kernel function.

Rotating the *xoy* coordinate system $\theta_{k}$ angle can get from (4).


$$\begin{array}{ccc} \begin{array}{c} u=R_{\theta_{k}}\left(\begin{array}{c} x\\ y \end{array}\right) \end{array} & & \end{array}$$ (4)

$R_{\theta_{k}}$ represents the rotation matrix with direction $\theta_{k}$, and Rθk= (cos ⁡ θksin ⁡ θk−sin ⁡ θkcos ⁡ θk),θk=(k−1)Kπ, *k* = 1 , 2 , ⋯ , *K* .  is the number of directions selected by the filter. Substituting Eq (4) into formula (3), a set of anisotropic Gaussian kernel functions can be obtained.

### 2.2 Anisotropic morphological Gaussian Kernel directional derivative

The early Prewitt gradient operator and the Canny operator can represent the anisotropic directional derivative (ANDD) in conjunction with the general rotating double window structure, as expressed in Eqs (5)–(9).


$$\begin{array}{ccc} \begin{array}{c} \nabla_{ANDD}^{\sigma ,\rho }(n)=iG_{h}(n)+jG_{v}(n) \end{array} & & \end{array}$$ (5)

$$\begin{array}{ccc} \begin{array}{c} G_{h}(n)=\underset{m_{x}>0}{\sum }w_{R,\sigma ,\rho }(n)x(n+m)-\underset{m_{x}<0}{\sum }w_{L,\sigma ,\rho }(n)x(n+m) \end{array} & & \end{array}$$ (6)

$$\begin{array}{ccc} \begin{array}{c} G_{h}(n)=\underset{m_{x}>0}{\sum }w_{R,\sigma ,\rho }(n)x(n+m)-\underset{m_{x}<0}{\sum }w_{L,\sigma ,\rho }(n)x(n+m) \end{array} & & \end{array}$$ (7)

$$\begin{array}{ccc} \begin{array}{c} w_{R,\sigma ,\rho }(n)=\frac{\rho \left|n_{x}\right|}{\sqrt{2\pi }\sigma^{3}}exp\left\{-\frac{1}{2\sigma^{2}}(\rho^{2}{\left(x+y\right)}^{2}+\rho^{-2}{\left(-x+y\right)}^{2})\right\}l(n_{x}\geq 0) \end{array} & & \end{array}$$ (8)

$$\begin{array}{ccc} \begin{array}{c} w_{L,\sigma ,\rho }(n)=\frac{\rho \left|n_{x}\right|}{\sqrt{2\pi }\sigma^{3}}exp\left\{-\frac{1}{2\sigma^{2}}(\rho^{2}{\left(x+y\right)}^{2}+\rho^{-2}{\left(-x+y\right)}^{2})\right\}l(n_{x}\leq 0) \end{array} & & \end{array}$$ (9)

In the above formula, *l*(*N*) is an indicator function that represents elements from a subset of the set *N*. $w_{R,\sigma ,\rho }(n)$ is defined as the double window function on the right half, and $w_{L,\sigma ,\rho }(n)$ is a left double window function. They are all non-negative functions, which are symmetrical about the longitudinal axis, their corresponding functions decay continuously and rapidly in a large range.

Considering the image details, the Weighted Median Filter (WMF) is capable of preserving significant information while effectively removing impulse noise [22]. A weighted median filter with a weight of *w*(*n*) that satisfies the can be defined as (10).


$$\begin{array}{ccc} WMF(Set(n)|w(n))\equiv median\{w(n)◇Set(n),n\in \Omega \} & & \end{array}$$ (10)

The vertical diamond symbol “ ◇ ” indicates repetitive operations, and the letter “median” denotes the median filtering. The three capitals *WMF* is the abbreviation for Weighted Median Filter.

The given fixed image *I*(*n*) combine the generate function $\left\{w_{R}(x),w_{L}(x)\right\}$ to express (11) the anisotropic morphological directional derivation of a certain pixel *n* in the image:


$$\begin{array}{ccc} \begin{array}{cc} \nabla_{AMDD}^{\sigma ,\rho }I(n|\theta )= & WMF(I(n+m)|w_{R,\sigma ,\rho }(R_{-\theta_{k}}m)) \end{array} & & \end{array}$$ (11)

$R_{-\theta_{k}}$ is the same as that in (4), and the range of *θ* belongs to  [0,2π). The bi-window $w_{R}(R_{-\theta_{k}}n)$ generated by the generating function of the right half-plane is numerically equal to the bi-window structure $w_{L}(R_{-(\theta_{k}+k)}n)$ of the left-half plane rotated by 180 degrees. Thus, it can be deduced that the AMDD response of the *θ* angle and the *θ*  ±  *π* direction are reciprocally opposite to each other.

## 3 Fusion edge map and a new edge detection method

This section analyzes the limitations of the Canny algorithm by examining the edge strength map (ESM), which is formed using a multi-scale approach combined with the anisotropic morphological Gaussian directional derivative, as well as the edge direction map (EDM) extracted through the AMDD directional matching filter. A new edge map is derived by combining the ESM and EDM using a specific strategy, which is then integrated into the conventional optimal Canny detector.

### 3.1 Expose the problems with the Canny algorithm

The Canny algorithm has several limitations and drawbacks. Firstly, Canny and other researchers employed a Gaussian filter with a scale parameter in the Gaussian function to mitigate the impact of noise. Generally, the accuracy of the edge will decrease as the scale *σ* increases, and the PSNR (peak signal-to-noise ratio) is directly proportional to the scale *σ*. Therefore, the appropriate scale *σ* value is a difficult problem for the classical Canny algorithm. Secondly, the use of mathematical partial derivatives is unavoidable in the process of calculating the gradient amplitude in the Canny algorithm, which can introduce noise and lead to false detections and missed edges. Additionally, in the standard Canny detection algorithm, the selection of double thresholds involves manually setting high and low values. As a result, some false edges may remain in the detection process. While the edge detection output may appear continuous at times, there can be significant variations in the quality of edge detection. Finally, the non-maximum suppression step is crucial for accurate edge extraction, even though it may result in the loss of true edges.

For the first three issues, there are currently no effective solutions; however, this paper will provide a thorough analysis of the fourth point. Whether it is an X-shaped intersection, a Y-type intersection, or a star-like intersection, the cross-point edges can be identified from the perspective of the Canny algorithm’s definition. However, these intersection points are often lost in practical detection results.

**Fig 1:**
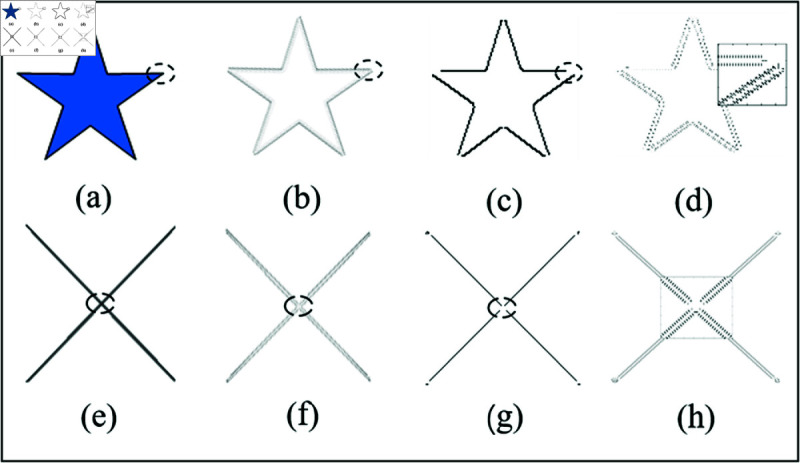
Results of Canny edge detector. (a) and (e) are the original images of the five-pointed star and the cross line, respectively, with the dotted line representing the cross edge. (b) and (f) correspond to images (a) and (e). (c) and (g) show edge results from the Canny detection algorithm, while (d) and (h) illustrate the gradient vector graphs of those results.

From the perspective of geometric deformation, the regions of the two input images in Fig 1 are homogeneous, with highly symmetrical gray-level information. The junctions of the edges in the image are shown in Fig 1(a) and 1(e). As illustrated in Fig 1(d) and 1(h), the gradient vector in the original image, which primarily concentrates on the edge junctions, is lost after edge detection by the Canny detector. Different types of edges require distinct considerations. Due to the non-maximum suppression step in Canny edge detection, the true edge pixels located at the edge intersections are suppressed. As shown in Fig 1(c) and 1(g), no edge signs are present at the junctions in the resulting image.

### 3.2 Discrete AMDD and fusion map

The image is still discrete, no matter how small the image is divided into grids and how much area each grid occupied. Therefore, the filter used should be discretized during the image processing. Let $u={\left[u_{x},u_{y}\right]}^{T}$ in the Cartesian coordinate system and then the discrete anisotropic Gaussian derivative can be defined as (12)


gσ,ρ,θk′(x)=−ux′cos ⁡ θk(ρ⋅σ)2[(ρ4+1)ux cos ⁡ θk+(ρ4−1)uy sin ⁡ θk]gσ,ρ,θk(x) (12)

Where, $\rho \geq 1,\sigma >0,\theta_{k}=\frac{\left(k-1\right)}{K}\pi ,k=1,2,\cdots \;,K$. The image is displayed on a grid, and the pixels represent the horizontal and vertical coordinates, so the seemingly continuous image can be discrete into each point. Similarly, a discrete form of anisotropic morphological Gaussian directional derivative matrix column can be constructed according to the discrete anisotropic Gaussian directional derivative expression (13):


$$\begin{array}{ccc} \begin{array}{c} AMDD\text{\_}M_{Discrete}\left\{I(n)\right\}\\ =\left[\begin{array}{cccc} \nabla_{AMDD}^{\sigma ,\rho }[I(n_{x}-1,n_{y}-1)|1] & \nabla_{AMDD}^{\sigma ,\rho }[I(n_{x}-1,n_{y})|1] & \cdots \; & \nabla_{AMDD}^{\sigma ,\rho }[I(n_{x}+1,n_{y}+1)|1]\\ \nabla_{AMDD}^{\sigma ,\rho }[I(n_{x}-1,n_{y}-1)|2] & \nabla_{AMDD}^{\sigma ,\rho }[I(n_{x}-1,n_{y})|2] & \cdots \; & \nabla_{AMDD}^{\sigma ,\rho }[I(n_{x}+1,n_{y}+1)|2]\\ \vdots & \vdots & \ddots & \vdots \\ \nabla_{AMDD}^{\sigma ,\rho }[I(n_{x}-1,n_{y}-1)|K] & \nabla_{AMDD}^{\sigma ,\rho }[I(n_{x}-1,n_{y})|K] & \cdots \; & \nabla_{AMDD}^{\sigma ,\rho }[I(n_{x}-1,n_{y}+1)|K] \end{array}\right]. \end{array} & & \end{array}$$ (13)

In the discrete AMDD expression (13), the capital M with the horizontal bar represents the initial letter of the matrix, and the discrete English term denotes the discrete matrix column of the AMDD.

For pixel $n=\left[\begin{array}{c} n_{x}\\ n_{y} \end{array}\right]$, a certain pixel range (such as a 3×3-pixel range) is selected to smooth the anisotropic morphological Gaussian directional derivative according to the theory of continuous image discretization. Thus the *K* directional derivatives is gained. Finally, a *K* × 9 AMDD matrix is constructed, in which the *K* is the number of directions and the number 9 is the pixel range.

The AMDD expression can be obtained by inputting the given image *I*(*n*). Then, the spatial response of the multi-directional AMDD $\nabla_{SMF}I(n|K)$ can be obtained through adopting the spatial matched filter $f_{k}^{\;SM}$. This is used to trace the edges of the AMDD image in K directions. As is shown in Eqs (14) and (15), the edge strength map (ESM) is derived based on the maximum AMDD amplitude response of the spatially matched filter across multiple pixels and in several directions.


$$\begin{array}{ccc} \begin{array}{ccc} ESM\left\{I(n|k)\right\}= & \underset{k}{max}\;\left\{\left|\nabla_{SMF}I(n|k)\right|\right\}=\underset{k}{max}\;\left\{\left|\underset{m}{\sum }f_{k}^{\;SM}(m)\nabla_{AMDD}^{\sigma_{k},\rho }I\left\{(n+m)|k\right\}\right|\right\} & \\ = & \underset{k}{max}\;\left\{\left|\underset{m,f_{k}^{\;SM}(m)=1}{\sum }\nabla_{AMDD}^{\sigma_{k},\rho }I\left\{(n+m)|k\right\}\right|\right\},k=0,1,\cdots \;,K. \end{array} & & \end{array}$$ (14)

$$\begin{array}{ccc} \begin{array}{c} f_{k}^{\;SM}(n)=\{\begin{array}{c} 1,\left|n_{x}\right|\leq (s+b∕2\;),n_{y}=round(n_{x},tg(\theta_{k}))\\ 0,otherwise \end{array},\theta_{k}\in \left[0,\frac{\pi }{4}\right]⋃\left[\frac{3}{4}\pi ,\pi \right),\\ f_{k}^{\;SM}(n)=\{\begin{array}{c} 1,\left|n_{y}\right|\leq (s+b∕2\;),n_{x}=round(n_{y},ctg(\theta_{k}))\\ 0,otherwise \end{array},\theta_{k}\in \left(\frac{\pi }{4},\frac{3\pi }{4}\right). \end{array} & & \end{array}$$ (15)

$\nabla_{SMF}I(n|k)$ denotes the product between k spatial matched filters $f_{k}^{\;SM}$ and the image AMDD amplitude response $\nabla_{AMDD}^{\sigma_{k},\rho }I(n|k)$, “b” is the width of bi-window and “s” is the distance between the two windows.

The EDM formula (16) will be obtained as follows. First, the image *I*(*n*) is input into a set of anisotropic morphological directional derivative filters, then receives different edge responses by adjusting the scale *σ* to three ranges of small, middle, and large. Finally, the maximum argument response is found through the scale product and fitting method.


EDM {I(n |k)}=arg ⁡ maxk [∏i=0k∇ ⁡AMDDσk,ρI(n |k)k],k=0,1,⋯,K. (16)

**Fig 2:**
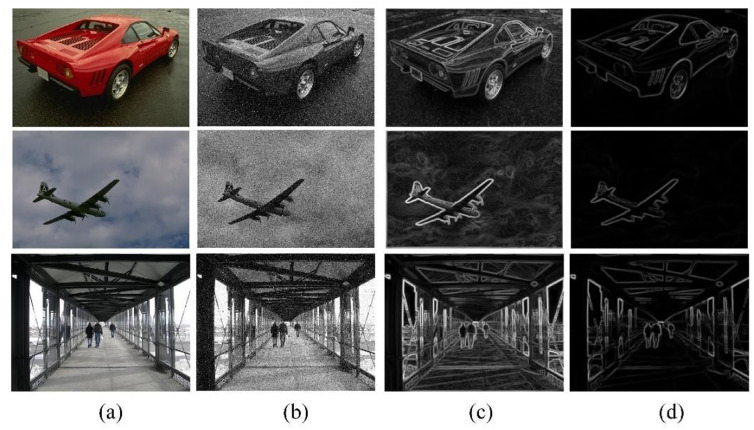
ESM and EDM of test images.

The spatially matched filter used in the process of capturing the ESM effectively enhances the accuracy of edge localization and improves the resolution of edge detection. In the acquisition of the EDM, the scale multiplication technique, achieved through multi-scale fusion of AMDD, reduces edge elongation effects and enhances the stability of the edge detector. As illustrated in Fig 2, three test images from different scenes are selected, and their corresponding ESM and EDM are obtained.

Fig 2(a) shows a scene diagram with three different orientations. Fig 2(b) presents the grayscale version of the test image, where the third pedestrian image is corrupted with Gaussian white noise (variance = 20) and salt-and-pepper noise (variance = 25.5). Image (c) in the third column is the ESM, and image (d) is the EDM corresponding to the same image. The newly obtained ESM and EDM are collectively referred to as fused edge maps.

### 3.3 A new method with edge detection

A new edge detection method is proposed, addressing the trade-off between noise robustness and edge resolution, as well as the limitations of the Canny algorithm. First, multi-scale automatic AMDD is employed to capture the local grayscale variation information from the input image, allowing for the extraction of the AMDD response expression. Next, a spatially matched filter is used to effectively localize edge positions in multiple directions, obtaining the maximum amplitude response at each pixel, which is termed the Edge Strength Map (ESM).

Furthermore, different scale parameters are adjusted to obtain varying AMDD responses. These responses are then fused using the scale product technique to extract the maximum response map, called the Edge Direction Map (EDM). Finally, the fusion of the ESM and EDM directly forms a new edge map, which can be embedded into the standard Canny detector (or the new ESM and EDM can be integrated separately into the Canny detector) to obtain the edge contours. This results in the multi-scale automatic anisotropic morphological Gaussian directional derivative (MAMDD) edge detection method.

## 4 Multi-scale anisotropic morphological Gaussian derivative edge detector

Considering the relationship between the signal-to-noise ratio (SNR), edge strength, and the standard deviation of noise, the SNR of the Gaussian directional derivative in multi-scale anisotropic morphology is analyzed. Furthermore, the performance of the MAMDD detector is enhanced by incorporating contrast equalization technology and a noise estimation method. As a result, a new MAMDD edge detector is proposed, and the edge detection process for this detector is outlined.

### 4.1 MAMDD signal-to-noise ratio compared with the automated anisotropy factor

It is assumed that the variance of the zero-mean white noise dB(x) is $\varepsilon_{dB}^{2}$ and an image is contaminated by it. Then, the capability of the anisotropic morphological directional derivative filter to suppress zero-mean white noise is the variance of the noise response is (17):


$$\begin{array}{ccc} \begin{array}{c} \varepsilon_{\hat{dB}}^{\text{2}}=E\left\{{\left(dB(x)*\nabla_{AMDD}^{\sigma_{k},\rho }\right)}^{2}\right\}=\iint {\left(\varepsilon_{dB}\cdot \nabla_{AMDD}^{\sigma_{k},\rho }\right)}^{2}dxdy=\frac{1}{2}{\left(\frac{\sigma }{\rho }\right)}^{2}\varepsilon_{\bar{dB}}^{2}=\frac{\varepsilon_{dB}^{2}}{4\sigma^{2}} \end{array} & & \end{array}$$ (17)

In this formula (17), $\varepsilon_{dB}$ refers to the standard deviation of zero-mean white noise, and $\varepsilon_{\bar{dB}}^{2}=E\left\{{\left(dB(x)*\nabla_{AMGK}^{\sigma_{k},\rho }\right)}^{2}\right\}=\frac{\rho^{2}\varepsilon_{dB}^{2}}{2\sigma^{4}}$ denotes the ability of an anisotropic morphological Gaussian kernel with a scale of *σ* to suppress zero-mean white noise.

It is simply to see that the noise response is inversely proportional to the square of the scale $\sigma^{2}$, which is directly proportional to the square of the zero-mean white noise variance $\varepsilon_{dB}^{2}$ and to the square of the ratio of anisotropic factor *ρ* to the scale *σ*, under certain circumstances. According to experience, *σ* = 5, $\rho =\sqrt{1.5}$ and $\theta_{k}=3∕4\;\pi$ are generally taken, to better achieve edge extraction accuracy and suppress image noise efficiently [23].

By introducing the Heaviside function, a step edge is expressed as:


Sα(x)=eH ( [sin ⁡ α,cos ⁡ α]x). (18)

Thereinto, the function *H*(*x*) satisfies: *x* ≥ 0, *H* ( *x* ) = 1; *x*<0, *H* ( *x* ) = 0, *e* represents an edge intensity value. In light of Eqs (17) and (18), for a step edge and AMDD filter whose direction is $\alpha +\frac{\pi }{2}$, the SNR of the edge response is expressed as:


SNRstepσk,ρ(x |θk)=sign (cos ⁡  (θk−α))⋅eεdB^φ {r− [cos ⁡ α,sin ⁡ α]xd1∕2 (θk−α,wR)} (19)

In the formula (15), $\varphi \left\{x\right\}=\{\begin{array}{c} 0,x\notin \left[-1,1\right],\\ 1,x\in \left(-1,1\right). \end{array}$ and *r* is the distance from the origin to the edge pixel, *sign*(*x*) is the symbol function, $d_{1∕2\;}\left(\theta_{k}-\alpha ,w_{R}\right)$ is the unique solution of the equation DR,β(d)≡∬x>0,(x cos ⁡ β+y sin ⁡ β)>dwR(y)dy=0.5, $\left(0\leq \beta \leq \frac{\pi }{2}\right)$, the variable *d* indicates the distance from the point *x* to the edge, $\theta_{k}=\frac{\left(k-1\right)}{K}\pi ,k=1,2,\cdots \;,K.$

SNR is defined as the ratio between the maximum magnitude of the AMDD response and the noise standard deviation $\varepsilon_{\hat{dB}}$ as is shown from the above formula (20) when the variable quantity $\alpha =\theta_{k}$, and the edge pixel *x* along the direction *θ* satisfies r− [cos ⁡ α,sin ⁡ α]x=0, thus the ratio of the maximum amplitude of AMDD response to the noise standard deviation $\varepsilon_{\hat{dB}}$SNR is $2\sigma e∕\varepsilon_{dB}$. The SNR of AMDD increased with the edge intensity *e* and the scale *σ*, decreasing with the increase of noise standard deviation $\varepsilon_{\hat{dB}}$, and having independent on the anisotropic factor *ρ*.

To highlight the edge resolution and positioning accuracy of MAMDD well, a resolution constant is used (20):


Cσ,ρ(θk)=2σ (ρ2sin ⁡ 2(θk)+ρ−2cos ⁡ 2(θk)). (20)

The larger the resolution constant value, the lower the accuracy of edge positioning and the resolution ratio; conversely, the smaller the resolution constant value, the higher the accuracy. According to the standard of Canny edge detection, noise stability is regarded as the maximum function of SNR for a given scale *σ* and direction number *K*. However, through the ratio of the SNR of the maximization and minimization function to the resolution constant, the automated anisotropic factor can be calculated (21).


ρautomatic(k)=argmaxρ {SNRstepσ,ρ(x |k)Cσ,ρ(k)}=argmaxρ {sign (cos ⁡  (π2k))e2σ (ρ2sin ⁡ 2 (π2k)+ρ−2cos ⁡ 2 (π2k))εdB^φ {r−[cos ⁡ α,sin ⁡ α]xd1∕2(π2k,wR)}}. (21)

When $\rho_{automatic}=1$ in Eq (17), it is deemed to be an isotropic Gaussian kernel factor. However, *k* = 8, and $\rho_{automatic}\approx 3.1864$. The default direction selection number is set to 8 to capture enough variational information in the gray image. In the process of the experiment, the parameter *σ* is always greater or equal to *ρ*, which is to ensure the feature that the Gaussian kernel and orientation derivative filter can inherit continuity as much as possible.

**Fig 3:**
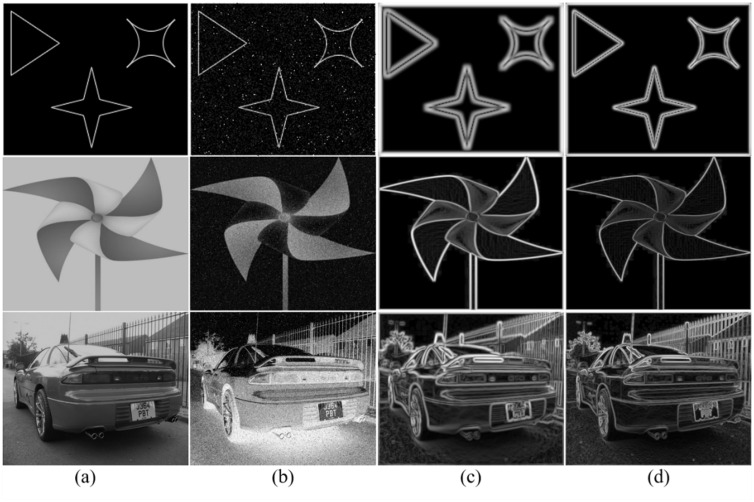
ESM of noise image. (a)Original image; (b) Image with a noise variance of 25.5; (c) ESM based on an isotropic Gaussian kernel; (d) ESM with an automatic anisotropic factor.

To explore the differences between automated anisotropic and isotropic factors, three experimental images are selected, each containing mixed noise and varying parameter characteristics to obtain the ESM under different scenarios. As shown in Fig 3.

As illustrated in Fig 3(a), three original test images are shown. In Fig 3(b), to evaluate the detector’s noise resistance, Gaussian white noise and salt-and-pepper noise with a variance of 25.5 are added to assess the anti-interference performance. Fig 3(c) presents the ESM obtained using the traditional isotropic Gaussian kernel, which exhibits noticeable edge blurring. In contrast, Fig 3(d) shows the ESM based on the automatic anisotropic factor. By comparison, it can be observed that the latter ESM is more stable to noise, with the extracted edges being clearly visible.

### 4.2 Noise estimation and contrast equalization

The image is often corrupted by various types of noise during the generation and transmission processes, which can significantly degrade image quality and adversely affect visual perception. Common types of noise encountered in images include Gaussian noise, impulse noise, Gamma noise, and uniform noise, all of which can disturb the image and reduce its overall quality. Noise-induced false edges in images can generally be classified into two categories. The ESM (edge-strength map) results from large gray-level fluctuations caused by noise and the edge blurring and stretching effects inherent to the ESM itself. The appearance of these false edges is primarily due to the lack of an actual edge in the affected pixel area; however, noise causes substantial pixel amplitude fluctuations in this region, leading to spurious edge formation. However, these areas originate from noise disturbance, which can be controlled by low-threshold operation of noise, and another type of false edge needs to be diminished by the precise value of the ESM design.

The cumulative distribution function (CDF) is introduced to fully characterize the probability distribution and occurrence of noise variables, as shown in Eq (22).


$$\begin{array}{ccc} F_{\sigma ,\mu }(x)=\frac{1}{\sigma \sqrt{2\pi }}\int_{-\infty }^{x}exp\left(\frac{-{\left(t-\mu \right)}^{2}}{2\sigma^{2}}\right)dt. & & \end{array}$$ (22)

Supposing the CDF of ESM of the unit noise variance is $F_{dB0}(x)$, and the variance of zero-mean Gaussian white noise *dB*(*x*) denotes $\varepsilon_{dB}^{2}$. Without loss of generality, it can be proved that the noise CDF with variance $\varepsilon_{dB}^{2}$, $F_{dB}(x)$ satisfies $F_{dB}(x)=F_{dB0}(\varepsilon_{dB}^{-1}x)$. For the first type of spurious edge, the probability of occurrence with their pixels represents $P_{f}$, and then the decision threshold is simplified as:


$$\begin{array}{ccc} T_{l}(P_{f})=\varepsilon_{dB}F_{dB0}^{-1}(1-P_{f}). & & \end{array}$$ (23)

**Fig 4:**
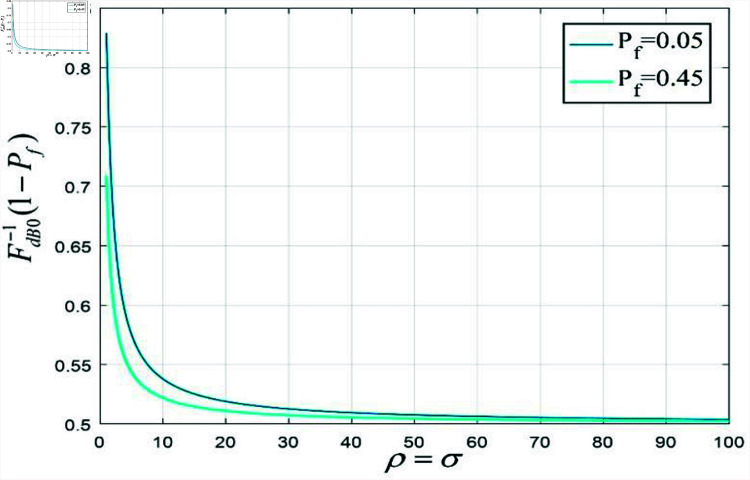
$F_{dB0}^{-1}(1-P_{f})$ curves with different anisotropic factors.

The $F_{dB0}^{-1}$ in the threshold expression (19) refers to the inverse function of the CDF function of the ESM unit noise variance. A series of non-linear operations are involved in calculating the ESM of unit variance noise, so the expression of CDF $F_{dB0}(x)$ cannot be gained. As is shown in Fig 4, a hundred points are selected in the interval of  [ 1,100  ]  to .form two curves of $F_{dB0}^{-1}(1-P_{f})$ function with satisfying $\sigma^{2}=\rho^{2}$, $P_{f}=0.05$ and $P_{f}=0.45$.

In several points in Fig 4, this specific pair of prime numbers was selected for the experiment to determine the significance of certain point values, as shown in Table 1.

**Table 1:** Several $F_{dB0}^{-1}(1-P_{f})$ values of different anisotropic factors.

$x\rho^{2}=\sigma^{2}$	1	3	5	7	11	13	17
$F_{dB0}^{-1}(0.95)$	0.8289	0.7083	0.6645	0.6402	0.6127	0.6039	0.5911
$F_{dB0}^{-1}(0.55)$	0.7088	0.6246	0.5971	0.5823	0.5659	0.5606	0.5531

As is shown in above, the threshold is proportional to the anisotropic factor when the alarm probability of the first type of spurious edge $P_{f}$ is fixed. With the increase of $\sigma^{2}=\rho^{2}$, the ability of the fused ESM to suppress noise is improving while the probability of losing the true edge is dwindling.

For the second type of false edge, this paper employs image contrast equalization to mitigate its effects. In certain regions of the image with relatively coarse texture details, some pixel edges correspond to large ESM values, but these do not represent true edges. In contrast, the ESM value at the boundary of an area with minimal grayscale variation is small, indicating a true edge pixel.

For a given image, its average variation can be shown as (24).


$$\begin{array}{ccc} \bar{s}=\frac{1}{MN}\underset{n}{\sum }\left\{ESM\left\{I(n|k)\right\}\times EDM\left\{I(n|k)\right\}\right\}. & & \end{array}$$ (24)

In above formula, M represents the length of the input image and N represents the width of the image. Additionally, the grayscale variation in areas containing edge pixels often differs from that in non-edge pixels. The variation in regions with fine detail and texture is generally more pronounced compared to that in flat areas.

For pixel *n*, the expression of the locally average variation is measured by:


$$\begin{array}{ccc} {\bar{s}}_{Local}(n)=\frac{1}{W}\underset{\tau \in W}{\sum }\left\{ESM\left\{I\left\{(n+\tau )|k\right\}\right\}\times EDM\left\{I\left\{(n+\tau )|k\right\}\right\}\right\}. & & \end{array}$$ (25)

*W* is a window centered on the origin in formula (25), and *τ* is the distance change value in the window. According to the machine vision system, changing the pixel value is adopted in edge detection, and the contrast equalization method is presented to improve the fusion of ESM and EDM:


$$\begin{array}{ccc} EM(n)=\frac{ESM\left\{I(n|k)\right\}\times EDM\left\{I(n|k)\right\}}{\bar{s}+\gamma {\bar{s}}_{Local}(n)}. & & \end{array}$$ (26)

The *γ* in Eq (26) is a constant, which is a compromise between the absolute magnitude and the relative amplitude. When the value of *γ* is equal to or smaller than 0, this value matches the absolute magnitude; otherwise, it is equivalent to the relative amplitude value.

### 4.3 MAMDD detector process

**Fig 5:**
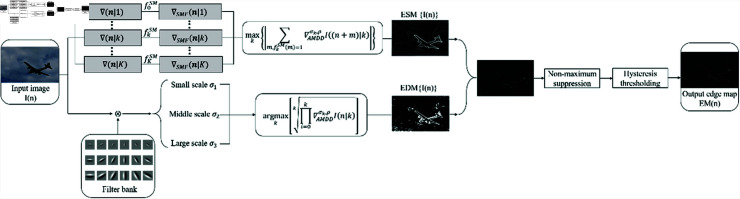
Flow chart of the proposed edge detection method.

For a set of parameters, *σ*, *ρ*, and *P* are assumed. There is an image *I*(*n*) corrupted by noise with a variance of $\varepsilon_{dB}^{2}$ and the anisotropic morphological Gaussian directional derivative and a couple of discrete anisotropic morphological directional derivative filters are obtained through the Eqs (16) and (12). This paper proposed the flow chart (Fig 5) of the multi-scale automated anisotropic morphological directional derivative MAMDD edge detector.

The figure above gives the specific edge detection process:

(I) The edge strength map (ESM) and the multi-scale edge direction map (EDM) are obtained separately and subsequently fused into a new edge map (EM) using the fitting product method. The fusion effect improves when the edge resolution constants are closer in value.

(II) Contrast equalization: For the second type of false edge, an improved edge map can be obtained through the computation of the average pixel change $\bar{s}$ and the local average variation ${\bar{s}}_{Local}$ of the image.

(III) Non-maximum suppression: the set of edge pixels, described by $\Lambda_{max}$, is formed through the way that each pixel in the given image, in which the modulus and gradient direction of the edge mapping ESM are used to determine the peak value of the gradient amplitude, thus forming a set of maximum points. The essence of non-maximum suppression is to identify the point with the enormous amplitude response on the edge intensity map to refine the edges and achieve the effect of thinning thick lines.

(IV) High and low thresholds: Hysteresis processing, the most critical step in edge detection, is inseparable from the high and low thresholds. Generally, the value of the high threshold is determined by the percentage of the improving EM histogram. Without the influence of noise, the values of the high threshold $T_{h}$ and the initial low threshold ${\tilde{T}}_{l}$ can be solved by the following formula (27).


$$\begin{array}{ccc} \begin{array}{c} \mathbb{R}n:\Lambda_{max}(n)<T_{h}=\delta_{h}MN\\ \mathbb{R}n:\Lambda_{max}(n)<{\tilde{T}}_{l}=\delta_{l}MN \end{array} & & \end{array}$$ (27)

Wherein *ℝ* represents the base of the set, and the value of $\delta_{h}$ is between [0.5,1], $\delta_{l}=0.4\delta_{h}$. If the image is polluted by noise, the low threshold value depends on the occurrence probability of the first type of false edge and the size of the noise standard deviation $\varepsilon_{dB}$, as shown (28).


$$\begin{array}{ccc} T_{l}(n)=max\left\{T_{l},\frac{\varepsilon_{dB}F_{dB0}^{-1}(1-P_{f})}{\bar{s}+\gamma {\bar{s}}_{Local}(n)}\right\}. & & \end{array}$$ (28)

The low threshold is related to noise stability and depends on spatial variation because the contrast equalization method affects the stability of the noise response.

(V) Hysteresis decision: The last step is determining edge pixels, which has two steps. By comparing the maximum suppressed set with the high threshold $T_{h}$, the set is larger than the threshold value filtered, which are named strong edge pixels $S_{edge}\equiv \{n:(\Lambda (n)\in \Lambda_{max}(n))\geq T_{h}\}$. Given a set $\mathbb{Z}\equiv \{n:T_{l}\leq (\Lambda (n)\in \Lambda_{max}(n))<T_{h}\}$, if a pixel in the set has a path to connect with a strong edge pixel according to the field criteria (usually four 4 Adjoining Points or 8 Adjoining Points), it is determined that the pixel in the set is an edge pixel. Similarly, these pixels are called a weak edge pixel set $W_{edge}$. In this way, the edge pixel map is composed of. In this way, the edge pixel map is composed of $S_{edge}$ and $W_{edge}$.

## 5 Experimental evaluation and results

This chapter mainly uses various evaluation indicators to evaluate the proposed edge detection algorithm. It proposed the algorithm and the six most advanced algorithms, regardless of the presence or absence of noise, which are compared and evaluated by using the accuracy recall (PR) curve and the quality factor (FOM) based on experiments [24].

The experiment of edge detection accuracy and noise stability is based on three data sets. It is the classic BSDS500 data set [25], the NYUDv2 data set in-depth learning, and the challenging PASCAL VOC 2007 data set [26], While the first two have corresponding GT images.

### 5.1 Evaluation index

In the field of image processing, numerous evaluation metrics have been proposed, including PR curve, ROC curve, FODS, FOIS, AP, R50 index, PSNR value, FOM index, and EEME (evaluation of entropy image enhancement) [27,28], among others. However, the FOM index and PR curve evaluation, which include $F_{ODS}$, $F_{OIS}$, AP, R50, and another index, are commonly utilized in edge detection.

**1. PR curve evaluation index**

The PR curve is an indicator that emphasizes sensitivity and specificity. It is constructed by fitting a series of corresponding sensitivity and specificity points obtained by setting the threshold values of different continuous variables. Generally, the larger the area under the curve, the higher the accuracy.

In binary classification, the points on the PR curve are calculated based on two instances: a positive class instance and a negative class instance. Ignoring repetition, these two examples have four possible outcomes, as determined by mathematical permutations and combinations. When a positive instance is classified as a positive class, it is considered a true positive (TP); when a positive instance is classified as a negative class, it is a false negative (FN). Similarly, when a negative instance is classified as a negative class, it is considered a true negative (TN), and when a negative instance is classified as a positive class, it is a false positive (FP). TP represents the number of correctly identified edge points, while FN refers to the number of false negatives or incorrectly identified points. From a broader perspective, TN represents the number of correctly identified non-edge points, and FP denotes the number of edge points that are incorrectly classified.

It is far from enough to get these variables to evaluate the quality of an edge detection method, so the correct rate, also known as the accuracy rate, does require to be introduced. It is computed by *P* = *TP* ∕ ( *TP* + *FP* ) . In addition, the true positive rate, also called sensitivity or recall rate, is calculated by the value of TP and FN *TPR* = *TP* ∕ ( *TP* + *FN* ) . The value reflects the probability that the identified positive instances account for all positive instances. However, it is no wonder that there is a false positive rate if there is a true positive rate, which corresponds to the equation *FPR* = *FP* ∕ ( *FP* + *TN* ) or*FPR* = 1 − *TN* ∕ ( *FP* + *TN* ) . The *TN* ∕ ( *FP* + *TN* )  is known as the true negative rate or true yin rate. However, the true positive rate reflects the probability that the negative instances wrongly considered to belong to the positive category account for all negative instances. So, the graph with the horizontal axis is TPR, and the vertical axis is P, which is the PR curve. Ideally, the result will be better if the point is closer to (0,1)(TPR=0, P=1) on the graph. But an ROC curve opposite to PR slightly is an image drawn with FPR recall as abscissa and TPR accuracy as ordinate.

**2. F-Measure and FOM index**

An indicator built on precision and recall is F-Measure [29], which is reckoned by their weighted harmonic average. The value of F-Measure can be given after getting the values of the precision rate P and the recall rate TPR, as follow as (29)


$$\begin{array}{ccc} F=\frac{(\lambda^{2}+1)P\cdot TPR}{\lambda^{2}P+TPR}. & & \end{array}$$ (29)

Generally, the value of parameter *λ* is 1. F-Measure can be used to evaluate a model, which also can be subdivided according to different threshold applications.

If a fixed threshold is selected to apply to all images in the database, the threshold is called the global optimal ODS (representing the best result obtained by using the same threshold in the database). Conversely, if each image has chosen one threshold only, this threshold is called the best OIS for a single image (describing the result acquired by utilizing the corresponding optimal threshold for each image). These evaluation indexes can be calculated during the experiment, associating them with F-Measure is $F_{ODS}$ and $F_{OIS}$ (denoting as F-Measure in cases of ODS and OI). R50 means the value of the accuracy rate corresponded to the region above the TPR curve at fifty percent of the TPR abscissa [29]. These evaluation indicators can all be calculated during the experiment.

When the training image does not have a corresponding ground truth (GT) image, the PR curve is replaced by the quality factor (FOM) proposed by Pratt as (30). The FOM for the edge map is defined based on three elements: false edges, positioning errors, and edge loss.


$$\begin{array}{ccc} FOM=\frac{1}{Max\left(N_{e},N_{d}\right)}\sum_{i=1}^{N_{d}}\frac{1}{1+vd^{2}(i)}. & & \end{array}$$ (30)

*ν* represents the loss factor of the position deviation from the edge pixel, takes a value of 0.25 generally, in which Ne indicates the number of edge pixels in the ideal edge map, $N_{d}$ is the number of edge pixels detected, and d(i) is the distance from the first detected pixel to the ideal edge pixel position. FOM=1 indicates an ideal situation.

**Remark 1**: It should be emphasized that in this study we used PR curves and FOM as the primary evaluation metrics rather than SSIM and RMSE most notably for several reasons including the following. First, PR curves and FOM are metrics that are widely recognized and established in edge detection research because they capture key performance aspects (PR curves evaluate the trade-off between precision and recall, providing insights into how algorithms balance false positives and false negatives in the edge detection task; FOM metrics are specifically tailored for edge detection to evaluate edge localization precision and robustness to noise) robustness. (It effectively quantifies the alignment between detected edges and ground truth edges, making it particularly suitable for comparing performance under different noise levels.) We believe that these metrics comprehensively capture the algorithm’s edge detection capabilities, including its ability to maintain high resolution, accuracy, and robustness to noise. While SSIM and RMSE are valuable metrics for evaluating image quality and pixel similarity, they are not typically used as primary metrics for edge detection evaluation. This is because 1. SSIM measures the structural similarity of the entire image. While it is useful for assessing image quality, it may not provide specific insights into the accuracy or localization of detected edges; 2. RMSE evaluates pixel differences, which may include uncorrelated non-edge regions, which may reduce its relevance in specific edge tasks. Our principle in the selection of evaluation metrics is to be able to most intuitively express the performance of the method proposed in this paper, so we chose the PR curve and FOM, and their performances fully illustrate the superiority of the method proposed in this paper.

### 5.2 Comparative evaluation

Experts typically delineate the ground truth (GT) image manually as a reference for comparative evaluation. It not only includes the edge regions that need to be detected, but also the areas that should not be considered as edges. In this paper, the proposed edge detector is compared with six state-of-the-art edge detectors (AAGK, Canny, IAGK, AMDD, MAGK, RCN), all of which utilize Gaussian filters to extract gray level change information for edge detection, but differ in their binarization strategies.

For the PR curve evaluation, if the distance between a detected edge pixel and the corresponding edge pixel in the GT image is within a specified tolerance of three pixels, it is considered a true positive (TP). If the distance exceeds this tolerance, the pixel is counted as a false positive (FP). The precision and recall rates are then calculated based on TP and FP. Since each detector has adjustable parameters, it is necessary to compare the performance of different detectors under specific parameter settings.

To plot the PR curve, the BSDS500 dataset is used, and ten object image sets and five aerial image sets are selected for training and testing. For calculating the PR curve, a unified threshold range is applied to all seven detectors, with $T_{l}=$ 0.01,0.02,…, 0.4, $T_{h}=$ 0.41,0.42,…, 0.99.

**Fig 6:**
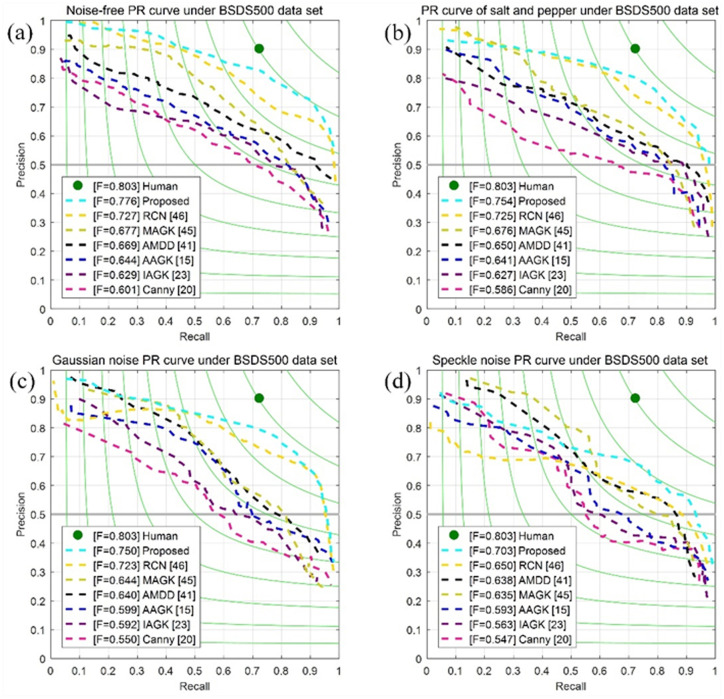
Comparison of comprehensive PR curves of seven detectors for ten images. Figure (a) is a noiseless PR curve, and Figure (c–d) is a PR curve corresponding to 5 variances of salt and pepper, ten variances of Gaussian, and 15 variances of speckle noise.

The only parameter of the Canny detector [22] scale is taken as $\sqrt{\text{2}}$. The three parameters of AAGK [16], kernel direction number, scale, and anisotropic factor take the values 8, $\sqrt{\text{7}}$ and $\sqrt{\text{7}}$ respectively. Similarly, the scale direction number and anisotropic factor of IAGK take the values $\text{2}\sqrt{\text{2}}$, 16 and $\text{2}\sqrt{\text{2}}$. The three scale values of MAGK can be selected in the range of [1,6], and the general values are 1, $\sqrt{\text{3}}$, $\sqrt{\text{6}}$, while the number of scales and directions are $\sqrt{\text{1}\text{.5}}$ and 8. And the parameter scale and anisotropic factor of AMDD are equal to $\sqrt{\text{6}}$. RCN uses an edge detection model, utilizing an optimization algorithm of non-maximum suppression for the extracted feature map. The proposed detector direction number is 16, and the automatic anisotropic factor and scale are $\sqrt{\text{7}}$. In Fig 6, the picture, combined with the selection of parameters, illustrates the PR curves of seven detectors under different ratios of noise.

**Fig 7:**
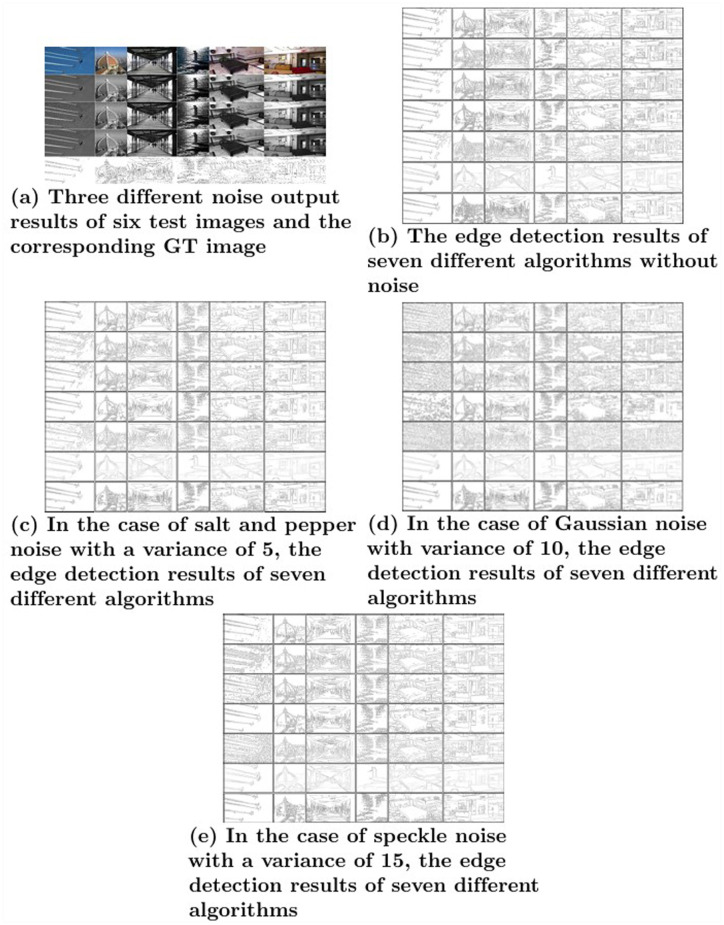
Experimental results.

In the absence of noise, the proposed detector slightly outperforms the RCN detector and is clearly distinguishable from the Canny detector, as shown in Fig 7. However, in the presence of salt-and-pepper, Gaussian, and speckle noise, the proposed detector demonstrates significant performance advantages, which become increasingly pronounced as the noise level decreases. Overall, the Canny detector is less effective, primarily due to its loss of edge resolution. In comparison, other detectors perform at a mid-level, while the proposed detector achieves optimal performance.

**Fig 8:**
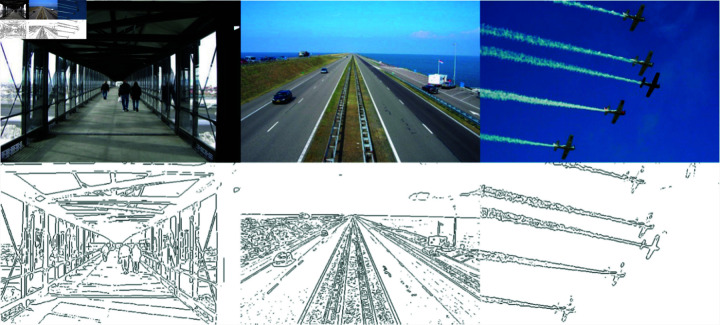
Three test images and corresponding GT images.

For the FOM index calculation, as shown in Fig 8 three test images (including images of both space and objects which do not have GT images) are selected from the PASCAL VOC 2007 dataset to obtain the FOM values for the seven detectors discussed in this paper.

In this study, following a similar approach as in previous works, the seven detectors are used for rule fusion to create a candidate edge set.

The proposed detector in FOM calculation is identical to the other six parameters selected, except for the amount of threshold variation, namely K=16, $\sigma^{2}=\rho^{2}=49$. For better comparison, lometric sampling was performed within [0.65,0.65,0.95] at 0.002 intervals, and finally the maximum FOM value was calculated according to Eq (27). Besides, using the same parameter and type noise with different numerical variances, the FOM value and the final mean can be calculated. It presents the spot noise data control with a variance of 5 and a Gaussian with a variance of 15 in Table 2.

Table 2 shows the FOM values of seven detectors under different types of noise. The first column corresponds to test images with salt-and-pepper noise, having a variance of 5. The second column presents test images with Gaussian noise, having a variance of 10. The final column shows test images with speckle noise, having a variance of 15.

The diagram above illustrates that the Canny and M. Canny detectors are significantly affected by salt-and-pepper or speckle noise. This is because the isotropic Gaussian kernel fails to resolve the trade-off between accurate edge localization and noise stability, and is limited by the choice of parameters. In contrast, the proposed detector demonstrates remarkable detection performance under varying levels of noise interference, particularly Gaussian noise. The performance of other detectors is generally at a mid-level. For speckle noise, the proposed detector is not the best in all aspects, but it is only 0.01% behind the optimal detector. The RCN detector, which exhibits exceptional noise robustness, relies on optimized algorithms for non-maximum suppression (NMS), benefiting from its advanced filter bank and morphological denoising techniques.

### 5.3 Experimental results

This paper selects six representative original images from two datasets used in the PR evaluation and one dataset used in the FOM evaluation. The result graphs of the seven detectors are presented in Fig 7, showing performance under both noise-free conditions and in the presence of salt-and-pepper noise, Gaussian noise, and speckle noise, with variances of 5, 10, and 15, respectively.

Fig 7 presents the results of the seven edge detectors. Fig 7(a) shows the test images with salt-and-pepper, Gaussian, and speckle noise added, with variances of 5, 10, and 15, respectively. The last row represents the ground truth (GT) images. Fig 7(b) displays the detection results of the detectors in the absence of noise. Figs 7(c), 7(d), and 7(e) show the detection results under salt-and-pepper noise, Gaussian noise, and speckle noise, respectively.

The first row in Fig 7(a) shows six test images, while the second to fourth rows display the images with different levels of noise variance. The last row corresponds to the ground truth (GT) images for each test image. In Fig 7(b)–7(e), the results are arranged in chronological order, starting with the classic Canny detector, followed by IAGK, AAGK, AMDD, MAGK, RCN, and finally the proposed detector.

From Fig 7(b), the noise-free results demonstrate that the edge map generated by the proposed algorithm is well-extracted, capturing all the relevant edge points accurately. In the noise-free results of Fig 7(c)–7(e), the edge map produced by the proposed detector is detailed and performs exceptionally well, successfully detecting all the important edge points.

**Table 2:** The FOM values of seven detectors under different types of noise.

Image	Bridge			Highway			Aircraft		
NDEPs	179680	180530	179610	180890	182080	180940	163140	163910	163520
Noise	$\varepsilon_{d}B^{2}=5$	$\varepsilon_{d}B^{2}=10$	$\varepsilon_{d}B^{2}=15$	$\varepsilon_{d}B^{2}=5$	$\varepsilon_{d}B^{2}=10$	$\varepsilon_{d}B^{2}=15$	$\varepsilon_{d}B^{2}=5$	$\varepsilon_{d}B^{2}=10$	$\varepsilon_{d}B^{2}=15$
AAGK	0.9872	0.9838	0.9872	0.9881	0.9843	0.9863	0.9834	0.9872	0.9828
Canny	0.9863	0.9829	0.9860	0.9872	0.9838	0.9854	0.9824	0.9868	0.9820
IAGK	0.9869	0.9837	0.9869	0.9878	0.9840	0.9859	0.9829	0.9874	0.9830
AMDD	0.9874	0.9845	0.9862	0.9879	0.9846	0.9865	0.9829	0.9887	0.9822
MAGK	0.9877	0.9846	0.9873	0.9872	0.9847	0.9866	0.9854	0.9887	0.9827
RCN	0.9885	0.9883	0.9881	0.9884	0.9856	0.9873	0.9851	0.9890	0.9829
Proposed	0.9886	0.9861	0.9882	0.9887	0.9861	0.9881	0.9854	0.9895	0.9832

**Remark 2**: The proposed method utilizes a multi-scale framework to extract edge strength maps (ESM) and edge direction maps (EDM) on three different scales. Each scale provides complementary properties: the small-scale ESM/EDM achieves high edge resolution and accuracy but is sensitive to noise; the large-scale ESM/EDM effectively reduces noise but may exhibit significant edge elongation; and the intermediate scale balances resolution, accuracy, and noise robustness. By combining these scales through scale fitting techniques, the algorithm synthesizes an edge map (EM) that coordinates the strengths of all scales while minimizing their respective weaknesses. This ensures adaptability to different noise characteristics. In addition, the proposed method incorporates an automatic anisotropy factor that dynamically adapts the morphological Gaussian kernel according to local image features. This allows the algorithm to adapt to orientation variations and different noise levels in different datasets, preserving edge details while suppressing irrelevant signals. Robust and consistent performance across different datasets is verified by experiments on multiple datasets with different noise characteristics and image complexity. The proposed method consistently outperforms state-of-the-art detectors. Although the proposed method shows excellent overall performance, we recognize that it may pose challenges in tasks with highly textured regions with low contrast, extremely high noise levels, and sharp discontinuities in edge structure, and we will focus on the performance of these tasks in future research to improve the existing method.

## 6 Conclusion

This paper proposes a novel fusion edge detection algorithm that abstracts the edge strength map (ESM) through spatial response filtering and anisotropic morphology. Meanwhile, the edge direction map (EDM) is generated by selecting the maximum value from a set of spoke values derived from the anisotropic morphological expression. Finally, the ESM and EDM are fused to create the edge map (EM), incorporating an improved automatic anisotropy factor.

Based on the PR curve, FOM values, and other evaluation metrics, the proposed algorithm outperforms the six state-of-the-art edge detection algorithms. Results from different datasets further demonstrate that the algorithm offers high accuracy, low false positive rates, and strong noise robustness. Additionally, the proposed detector not only mitigates edge elongation to some extent but is also adaptable to various datasets.

## References

[1] Zhu Y, Gao Y, Wang M, Li M, Wang K. Implementation of resource-efficient fetal echocardiography detection algorithms in edge computing. PLoS One 2024;19(9):e0305250. doi: 10.1371/journal.pone.0305250 39312521 PMC11419364

[2] Dong B, Weng G, Bu Q, Zhu Z, Ni J. An active contour model based on shadow image and reflection edge for image segmentation. Exp Syst Appl. 2024;238:122330. doi: 10.1016/j.eswa.2023.122330

[3] Roberts LG. Machine perception of three-dimensional solids [phd]. Cambridge: Massachusetts Institute of Technology; 1963.

[4] Amsaveni A, Palanisamy S, Guizani S, Hamam H. Next-generation secure and reversible watermarking for medical images using hybrid Radon-Slantlet transform. Results Eng. 2024;24:103008. doi: 10.1016/j.rineng.2024.103008

[5] Sobel I. Camera models and machine perception; 1970. Available from: https://api.semanticscholar.org/CorpusID:62214755.

[6] Prewitt J. Object enhancement and extraction. Picture Process Psychopictorics. 1970;10(1):15–9.

[7] Sun Z, Feng Y, Guo P, Dong Z, Zhang J, Liu J. Flash-based in-memory computing for stochastic computing in image edge detection. J Semiconduct. 2023:44(5);054101.

[8] Lee J, Haralick R, Shapiro L. Morphologic edge detection. IFAC Proc Vol. 1986;19(9):7–14.

[9] Jin J, Zhou W, Yang R, Ye L, Yu L. Edge detection guide network for semantic segmentation of remote-sensing images. IEEE Geosci Remote Sens Lett. 2023;20:1–5. doi: 10.1109/lgrs.2023.3234257

[10] Soria X, Sappa A, Humanante P, Akbarinia A. Dense extreme inception network for edge detection. Pattern Recognit. 2023;139:109461.

[11] Song X, Neuvo Y. Robust edge detector based on morphological filters. In: Proceedings of the 1991 International Conference on Circuits and Systems. 1991;1:332–5.

[12] Yan S, Cai H, Wang Y, Lu D, Wang M. Infrared and visible image fusion based on semi-global weighted least squares and guided edge-aware filters. Optics Lasers Eng. 2024;183:108533. doi: 10.1016/j.optlaseng.2024.108533

[13] Sun R, Lei T, Chen Q, Wang Z, Du X, Zhao W, et al. Survey of image edge detection. Front Signal Process. 2022;2:826967. doi: 10.3389/frsip.2022.826967

[14] Liu L, Liu Z, Hou A, Qian X, Wang H. Adaptive edge detection of rebar thread head image based on improved Canny operator. IET Image Process 2023;18(5):1145–60. doi: 10.1049/ipr2.13015

[15] Williams I, Bowring N, Svoboda D. A performance evaluation of statistical tests for edge detection in textured images. Comput Vision Image Understand. 2014;122:115–30.

[16] Jing J, Liu S, Wang G, Zhang W, Sun C. Recent advances on image edge detection: a comprehensive review. Neurocomputing. 2022;503:259–71.

[17] Wei Q, Feng D, Xie H. Edge detector of SAR images using crater-shaped window with edge compensation strategy. IEEE Geosci Remote Sens Lett. 2016;13(1):38–42.

[18] Liu J, Li C, Pan J, Guo J. Retracted article: visual communication of moving images based on AI recognition and light sensing image edge detection algorithm. Opt Quant Electron. 2024;56(4). doi: 10.1007/s11082-024-06542-0

[19] Peng-Lang Shui, Fu-Ping Wang. Anti-impulse-noise edge detection via anisotropic morphological directional derivatives. IEEE Trans Image Process 2017;26(10):4962–77. doi: 10.1109/TIP.2017.2726190 28715330

[20] Vimala M, Palanisamy S, Guizani S, Hamam H. Efficient GDD feature approximation based brain tumour classification and survival analysis model using deep learning. Egypt Inf J. 2024;28:100577. doi: 10.1016/j.eij.2024.100577

[21] Venkatesan V, Panangian D, Reyes M, Bittner K. SyntStereo2Real: edge-aware GAN for remote sensing image-to-image translation while maintaining stereo constraint. arXiv preprint 2024

[22] Jing J, Liu S, Wang G, Zhang W, Sun C. Recent advances on image edge detection: a comprehensive review. Neurocomputing. 2022;503:259–71.

[23] Zhang W, Sun C. Corner detection using multi-directional structure tensor with multiple scales. Int J Comput Vis 2019;128(2):438–59. doi: 10.1007/s11263-019-01257-2

[24] Martin DR, Fowlkes CC, Malik J. Learning to detect natural image boundaries using local brightness, color, and texture cues. IEEE Trans Pattern Anal Mach Intell 2004;26(5):530–49. doi: 10.1109/TPAMI.2004.1273918 15460277

[25] Ghandorh H, Boulila W, Masood S, Koubaa A, Ahmed F, Ahmad J. Semantic segmentation and edge detection—Approach to road detection in very high resolution satellite images. Remote Sens. 2022:14(3);613.

[26] Song Y, Li C, Xiao S, Zhou Q, Xiao H. A parallel Canny edge detection algorithm based on OpenCL acceleration. PLoS One 2024;19(1):e0292345. doi: 10.1371/journal.pone.0292345 38180975 PMC10769061

[27] Li S, Shen Y, Wang Y, Zhang J, Li H, Zhang D, et al. PiDiNet-TIR: An improved edge detection algorithm for weakly textured thermal infrared images based on PiDiNet. Infrared Phys Technol. 2024;138:105257.

[28] Agaian SS, Silver B, Panetta KA. Transform coefficient histogram-based image enhancement algorithms using contrast entropy. IEEE Trans Image Process 2007;16(3):741–58. doi: 10.1109/tip.2006.888338 17357734

[29] Ghani A, Isa N. Enhancement of low quality underwater image through integrated global and local contrast correction. Appl Soft Comput. 2015;37:332–44.

